# Dose differences in intensity‐modulated radiotherapy plans calculated with pencil beam and Monte Carlo for lung SBRT

**DOI:** 10.1120/jacmp.v16i6.5514

**Published:** 2015-11-08

**Authors:** Han Liu, Tingliang Zhuang, Kevin Stephans, Gregory Videtic, Stephen Raithel, Toufik Djemil, Ping Xia

**Affiliations:** ^1^ Department of Radiation Oncology Cleveland Clinic Cleveland OH USA

**Keywords:** lung SBRT, IMRT, dose calculation, Monte Carlo, pencil beam

## Abstract

For patients with medically inoperable early‐stage non‐small cell lung cancer (NSCLC) treated with stereotactic body radiation therapy, early treatment plans were based on a simpler dose calculation algorithm, the pencil beam (PB) calculation. Because these patients had the longest treatment follow‐up, identifying dose differences between the PB calculated dose and Monte Carlo calculated dose is clinically important for understanding of treatment outcomes. Previous studies found significant dose differences between the PB dose calculation and more accurate dose calculation algorithms, such as convolution‐based or Monte Carlo (MC), mostly for three‐dimensional conformal radiotherapy (3D CRT) plans. The aim of this study is to investigate whether these observed dose differences also exist for intensity‐modulated radiotherapy (IMRT) plans for both centrally and peripherally located tumors. Seventy patients (35 central and 35 peripheral) were retrospectively selected for this study. The clinical IMRT plans that were initially calculated with the PB algorithm were recalculated with the MC algorithm. Among these paired plans, dosimetric parameters were compared for the targets and critical organs. When compared to MC calculation, PB calculation overestimated doses to the planning target volumes (PTVs) of central and peripheral tumors with different magnitudes. The doses to 95% of the central and peripheral PTVs were overestimated by 9.7%±5.6% and 12.0%±7.3%, respectively. This dose overestimation did not affect doses to the critical organs, such as the spinal cord and lung. In conclusion, for NSCLC treated with IMRT, dose differences between the PB and MC calculations were different from that of 3D CRT. No significant dose differences in critical organs were observed between the two calculations.

PACS number: 87.53.Ly

## I. INTRODUCTION

Stereotactic body radiation therapy (SBRT) has been increasingly used in the primary management of medically inoperable patients with early stage non‐small cell lung cancer (NSCLC). Numerous clinical reports have demonstrated excellent local control of the primary tumor with minimal normal tissue toxicity.^1^, ^2^, ^3^, ^4^, ^5^ An accurate correlation between the computed dose and local tumor control achieved by SBRT is clinically important. For patients treated with early SBRT plans using pencil beam calculation algorithm, identifying dose differences between the Pencil Beam (PB) and Monte Carlo (MC) dose calculation is important for understanding treatment outcomes because these patients have the longest follow ups.

In the past decade, mixed use of treatment technologies and dose calculation algorithms has been permitted in various cooperative group trials for lung SBRT. For example, Radiation Therapy Oncology Group (RTOG) 0236 for peripheral tumors, defined as a tumor located >2 cm beyond the trachea‐bronchial tree, used three‐dimensional conformal radiotherapy technique (3D CRT) and allowed a PB calculation algorithm without tissue heterogeneity correction.^(6)^ The RTOG 0813 trial for "centrally" located tumors, defined as either <2 cm from the trachea‐bronchial tree or adjacent to mediastinal or pericardial pleura,^(7)^ allowed treatment technique of 3D CRT or intensity‐modulated radiotherapy (IMRT) and permitted PB dose calculation, but required tissue heterogeneity correction.

Previous works have demonstrated dose differences in SBRT delivered with 3D CRT between the PB and other methods including the MC calculation,^8^, ^9^ and furthermore, our work has shown that the dose differences exhibit a strong tumor volume and location dependence.^(9)^ If IMRT is the technique employed in delivering the treatment, it is unclear whether involving small segments in IMRT plans would deviate the dose differences between the MC and PB calculation from those of 3D CRT plans as previously reported.^8^, ^9^ Recent work by Chetty et al.^(10)^ considered IMRT and 3D CRT plans, and analyzed dose differences between the PB calculations and a variety of other calculation methods; they also found tumor and volume dependence for 133 NSCLC patients treated using 48 Gy in 4 fractions. However, their study did not analyze the IMRT plans alone, and so the dosimetric differences between the PB and more accurate algorithms in IMRT plans could not be established. Moreover, because IMRT has potential as a planning technique for improved normal tissue sparing, more research is needed to characterize the dose differences between the PB and other algorithms in critical organs. The aim of the current study is to investigate whether the dose differences between the MC‐ and PB‐calculated plans in the setting of IMRT delivery are similar to the previously reported dose differences from 3D CRT plans for both peripheral and centrally located tumors and their critical normal organs.

## II. MATERIALS AND METHODS

This retrospective analysis included 70 (35 central and 35 peripheral) patients with early‐stage NSCLC treated with 50 Gy in 5 consecutive fractions from 2009 to 2013. All patient data were collected in an institutional review board–approved prospective registry. Patients were immobilized daily using a vacuum bag restriction system (BodyFIX, Medical Intelligence Medizintechnik GmbH, Schwabmünchen, Germany) at the simulation and daily treatment. Abdominal compression was used to restrict tumor motion to <1 cm under fluoroscopic guidance. Four‐dimensional CT (4D CT) and serial CT chest scans while free breathing, at full inspiration and full exhalation, were acquired. The internal target volume (ITV) was created from 4D CT or multiphase CTs, and the planning target volume (PTV) was created by a 5 mm uniform expansion of the ITV.

The original IMRT plans used for treatment involved six or seven coplanar 6 MV beams, with the PB calculation and tissue heterogeneity correction employed in the iPlan RT treatment planning system (Brainlab AG, Feldkirchen, Germany). Each clinical plan was normalized such that at least 95% of the PTV received the prescription dose, and more than 99% of the PTV received at least 90% of the prescription dose. The maximum point dose and dose‐volume constraints of several critical organs are listed in Table 1.^(7)^ All the patients were treated in Novalis (Brainlab AG) platform with daily Exactrac image guidance.

Both the MC and PB dose calculation algorithms were implemented in the iPlan TPS. The MC dose calculation was based on the X‐ray Voxel MC algorithm developed by Kawrakow and Fippel,^11^, ^12^ which consisted of three main components: source modeling, beam collimating system modeling, and patient dose computation (see Brainlab AG Technical Reference Guide for more details).

**Table 1:** Planning acceptance objectives for critical organs

*Serial Tissue*	*Volume*	*Volume Max. (Gy)*	*Max. Point (Gy)*
Spinal Cord	<0.25 cc	22.5	30
	<0.5 cc	13.5	
Ipsilateral Brachial Plexus	<3 cc	30	32
Esophagus	<5 cc	27.5	52.5
Heart	<15 cc	32	52.5
Trachea and Ipsilateral Bronchus	<4 cc	18	52.5

All clinical IMRT plans were recalculated with the MC algorithm with heterogeneity correction. Identical beam configurations and monitor unit settings were used in the recalculated plans. Parameters for the MC dose calculation were 2 mm for the spatial resolution, 2% for the mean variance, "dose to medium" for the dose result type, and "accuracy optimized" for the multileaf collimator (MLC) modeling.

Selected dose‐volume parameters were compared for the MC and PB calculations, including $D_{1}$ (dose to 1% of the volume, a representative of maximum dose), $D_{95}$ (dose to 95% of the volume), and $D_{99}$ (dose to 99% of the volume) of the ITV and PTV; mean lung dose (MLD); $V_{20}$ (lung volume that receives 20 Gy) for the combined lungs; and $D_{1}$ for all the other organs at risk (OARs) such as the spinal cord, brachial plexus, and trachea.

## III. RESULTS

For the central tumors, the average volumes were 37.4±31.0 cc (range from 1.15 cc to 108.5 cc) for the ITV, and 76.6±50.4 cc (range from 9.0 cc to 191.6 cc) for the PTV. For the peripheral tumors, the average volumes were 18.1±17.0 cc (range from 1.4 cc to 62.9 cc) for the ITV, and 46.4±31.9 cc (range from 7.8 cc to 127.2 cc) for the PTV. Table 2 lists the distribution of the tumor locations for the group of the patients included in this analysis.

**Figure 1:**
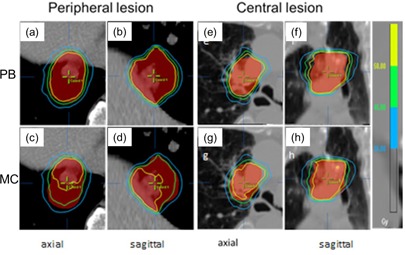
Comparison of isodose distributions between the PB ((a), (b), (e), (f)) and the MC ((c), (d), (g), (h)) dose calculations on the axial and sagittal isocentric slices for two typical patients.

**Figure 2:**
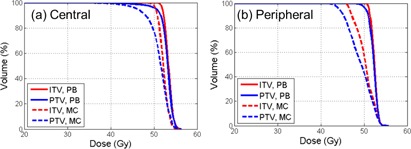
Comparison of DVHs of the target for the PB and MC calculations: (a) centrally located tumor; (b) peripherally located tumor.

As shown in the axial and sagittal images, Fig. 1 illustrates the dose differences in relationship to the tumor volume (PTV) with the MC and PB calculations for two selected patients (one peripheral and one central). The corresponding dose‐volume histograms (DVHs) of the PTV and ITV are shown in Fig. 2. For both patients, $D_{95}$ of the PTVs calculated with the PB method were >50 Gy. With the MC calculation, the $D_{95}$ was reduced to 46.1 Gy for the central tumor and 44.3 Gy for the peripheral tumor, demonstrating overestimation of the PTV dose by using the PB calculation.

**Figure 3:**
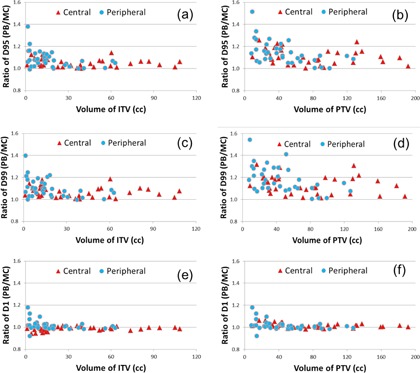
Ratio of $D_{1}$, $D_{95}$, and $D_{99}$ between the PB and MC dose calculations for the ITV and PTV: (a) $D_{95}$ of ITV; (b) $D_{95}$ of PTV; (c) $D_{99}$ of ITV; (d) $D_{99}$ of PTV; (e) $D_{1}$ of ITV; (f) $D_{1}$ of PTV

Figure 3 shows the ratio of $D_{95},D_{99}$, and $D_{1}$ between the PB and MC dose calculations as a function of the target volumes. With the MC calculation, $D_{95}$ and $D_{99}$ of the PTV for all patients were decreased. However, the ratios of $D_{1}$ of the PTV calculated from the PB and MC calculations fluctuated around 1. For small tumors (volume <40 cc), larger differences in $D_{95},D_{99}$, and $D_{1}$ of the PTV were observed. The average dose overestimation in $D_{95},D_{99}$, and $D_{1}$ of the ITV and PTV from the PB calculation is listed in Table 3 for both peripherally and centrally located tumors. Normalized to the MC calculation, the PB calculation showed significant dose overestimation in $D_{95}$ and $D_{99}$ of the ITV and PTV (p<<0.05 from the paired Student's *t*‐test). Differences in $D_{1}$ of the ITV and PTV between the PB and MC calculations were statistically significant, but were less than 2% (p<0.05, paired *t*‐test).

**Table 2:** Tumor locations of 70 SBRT patients

	*RUL*	*RLL*	*RML*	*LUL*	*LLL*
Central	10	7	2	11	5
Peripheral	9	7	2	12	7

LUL = left upper lobe; LLL = left lower lube; RUL = right upper lobe; RLL = right lower lube; RML = right middle lobe.

Dose overestimation in our dose‐volume parameters ($D_{95},D_{99}$, and D_1_) from the PB calculations were not significantly different between centrally and peripherally located tumors, and this held true for both the PTV and the ITV (two‐sample *t*‐test, unequal variance). The p‐values from the *t*‐test were 0.12, 0.05, and 0.85 for the $D_{95}$, $D_{95}$, and $D_{1}$ of the PTV, respectively. The corresponding p‐values for the ITV were 0.08, 0.05, and 0.75, respectively.

The ratios of $D_{95}$ and $D_{99}$ of the PTV between the PB and MC calculations showed large variations for the peripherally located small tumors (volume <40 cc, Fig. 3). For $D_{95}$ of the PTV, the average ratio for small tumors was 1.19±0.12, which was nearly 9% larger than the ratio for large tumors (p=0.009, *t*‐test with unequal variance). For $D_{99}$ of the PTV, the average ratio for small tumors was 1.23±0.12, which was nearly 9% larger than the ratio for large tumors (p=0.027, *t*‐test with unequal variance). Therefore, more severe dose overestimation by PB calculations occurred for the peripheral tumors with small volumes. However, no statistically significant volume dependence for dose overestimation from the PB calculations was observed in $D_{95}$ (p=0.027) and $D_{99}$ (p=0.22) of the PTV for centrally located tumors.

**Figure 4:**
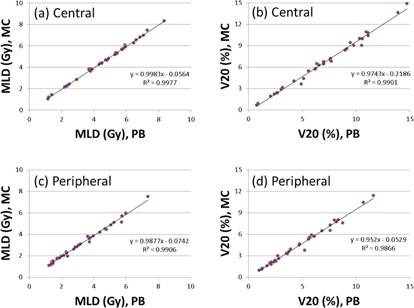
Correlations of the mean dose and $V_{20}$ of combined lungs for plans calculated with the PB and MC.

For the MLD and $V_{20}$ of the combined lungs, excellent correlations were observed between the MC and PB calculations (Fig. 4). For the central tumors, linear fittings resulted in the slopes of 0.998 and 0.974 for the MLD and $V_{20}$ of combined lungs, respectively. For the peripheral tumors, the corresponding slopes were 0.991 for the MLD and 0.952 for $V_{20}$ of the combined lungs. No significant differences were observed between the PB and MC calculations for the MLD. However, the PB calculation resulted in an average 5% overestimation of $V_{20}$ for the peripheral tumors and 2.5% for the central tumors when compared to the MC calculation.

**Table 3:** Average percentage difference in selected endpoint between the PB and MC

	*Peripheral*		*Central*	
	*ITV*	*PTV*	*ITV*	*PTV*
$\delta D_{95}\;(\%)$	7.1±6.0	12.0±7.3	5.1±3.5	9.7±5.6
$\delta D_{99}\;(\%)$	8.8±6.5	14.5±8.1	6.2±4.2	11.2±6.5
$\delta D_{1}\;(\%)$	1.4±2.0	1.5±1.9	1.7±4.3	1.7±4.0

The average variations in the MLD and $V_{20}$ for the combined lungs between the PB and MC calculations are listed in Table 4, together with the maximum dose differences for other OARs. From Table 4, the dose differences in $D_{1}$ of OARs were negligible between the PB and MC calculations for both centrally and peripherally located tumors. For both the PB and MC calculations, none of the maximum doses of OARs exceeded the dose constraints listed in Table 1.

**Table 4:** Selected endpoints of the organs at risk between the PB and MC

	δD	
*Parameters*	*Peripheral*	*Central*
$\left({\text{MLD}}_{\text{MC}}–{\text{MLD}}_{\text{PB}})\;(\text{Gy}\right)$	−0.11±0.15	−0.06±0.09
$\left(V_{20,\;\text{MC}}–V_{20,\;\text{PB}})(\%\right)$	−0.27±0.35	−0.40±0.36
$\left(D_{1,\text{MC}}-D_{1,\text{PB}})\text{of Cord}(\text{Gy}\right)$	0.12±0.28	0.16±0.27
$\left(D_{1,\text{MC}}-D_{1,\text{PB}})\text{of Esophagus}(\text{Gy}\right)$	0.13±0.35	0.11±0.39
$\left(D_{1,\text{MC}}-D_{1,\text{PB}})\text{of Brachial Plexus}(\text{Gy}\right)$	−0.01±0.70	0.02±0.25
$\left(D_{1,\text{MC}}-D_{1,\text{PB}})\text{of Heart}(\text{Gy}\right)$	−0.02±0.46	0.22±0.38
$\left(D_{1,\text{MC}}-D_{1,\text{PB}})\text{of Trachea}(\text{Gy}\right)$	−0.07±0.62	−0.22±0.82
$\left(D_{1,\text{MC}}-D_{1,\text{PB}})\text{of Proximal Bronchial Tree}(\text{Gy}\right)$	−0.25±0.68	−0.79±0.98

## IV. DISCUSSION

Treatment outcomes of radiotherapy are influenced by the planned dose. Early SBRT patients treated with PB calculated plans have longer clinical follow‐up, so identifying the dose differences between the PB predicted dose and Monte Carlo calculated dose for these patients is important in correlating the planned dose with the clinical outcome. The method of tissue heterogeneity correction and the dose calculation algorithm are two important factors that affect the dose accuracy in lung SBRT. Several investigators have reported that the MC algorithm in the iPlan improved the dose calculation accuracy when compared to the PB algorithm.^13^, ^14^, ^15^ In both homogeneous and heterogeneous phantoms, excellent agreements between the MC calculations and experimental measurements were reported in both high‐dose and high‐dose‐gradient regions. Comparisons of the MC calculated and measured percentage depth doses, dose profiles, and output factors for various field sizes in a uniform water phantom from our clinic were reported in our previous study.^(9)^

To better understand the treatment efficacy of SBRT for NSCLC, we recalculated the planned dose using the MC algorithm for 70 patients treated with IMRT and compared it with the clinical PB calculations. Our goal was to investigate whether the dosimetric differences between the MC and PB for IMRT plans were different from those of 3D CRT plans.

Using RTOG 0813 protocol as the plan acceptance criteria, 70 clinical plans calculated with the PB algorithm met criteria of the PTV coverage and minimal dose constraints ($V_{50\text{Gy}}>95\%$ and $D_{99}>45\text{Gy}$). However, with the PB dose calculation, $D_{95}$ and $D_{99}$ of the PTV were significantly overestimated, independent of tumor locations. These dosimetric differences can be attributed to the differences in modeling photon attenuations and lateral electron transportations between the PB and MC algorithms. In the PB algorithm, the tissue inhomogeneity is accounted for by applying a correction factor based on the equivalent length, while the lateral electron transport is ignored. The MC algorithm simulates individual interactions of photons and electrons with matter by using well‐established interaction probability distributions from experiments. A small but statistically significant overestimation of the maximum dose of the PTV was found in the PB calculation when compared to that from the MC calculation. In our previous study,^(9)^ we reported that the maximum dose was underestimated only by the PB calculation without tissue heterogeneity correction. The difference from this study and our previous study may be attributed to whether or not the tissue heterogeneity correction is applied.

Based on the MC calculation, the magnitudes of dose overestimation from the PB calculation were significantly volume‐dependent for the peripherally located tumors. The smaller the PTV volumes, the greater the dose overestimation found. This result agreed with the previous study.^(10)^ However, the observed tumor volume dependence in dose overestimation was not found in centrally located tumors. A future study with a larger sample size of patients is necessary to confirm our observation of the tumor volume independence for centrally located tumors. In contrast to previous studies,^(9)^ the ratios of $D_{1},D_{99}$, and $D_{1}$ of the PTV between the PB and MC calculations were similar for the centrally and peripherally located tumors, suggesting dose differences between the PB and MC calculations do not depend on tumor location for IMRT plans.

For all the organs at risk, differences in the maximum dose between the PB and MC calculations were negligible. Excellent correlations were observed for both the MLD and $V_{20}$ of combined lung between the PB and MC calculations. A simple rescaling can be applied to the volume/dose endpoints when considering radiation treatment related toxicity to the lungs. Previous studies reported that PB calculations overestimated MLD by 7% when compared to MC calculations for centrally located tumors,^(16)^ and by 5% for patients with the peripheral tumors.^(9)^ However, no significant differences were observed between the PB and MC calculations for the MLD in our patient samples. These different results may stem from the delivery methods. Our study included IMRT plans, while other studies included 3D conformal arc plans.^8^, ^9^

One limitation of this study is that it did not directly compare the IMRT SBRT plans to 3D CRT SBRT plans. Zhao et al.^(17)^ compared the dose calculation accuracy for conventional fractionation plans, directly comparing 3D CRT and IMRT plans for 24 patients. The results of their study cannot directly compare with our study, because of different tumor volumes. The average GTV and PTV from Zhao's study were 68.9±56 cc and 133.9±99.2 cc, respectively. For the central tumor of the present study, the average volumes were 37.4±31.0 cc for the ITV, and 76.6±50.4 cc for the PTV. For the peripheral tumors, the average volumes were 18.1±17.0 cc for the ITV, and 46.4±31.9 cc for the PTV. In Zhao's study, they showed that the dose deviations between the PB and Monte Carlo calculations in 3D CRT plans are different from the dose deviations in IMRT plans. Because a different photon energy (8 MV) was used in Zhao's study than the photon energy (6 MV) used in the present study, the reported dose differences cannot be directly compared to our results, either.

Despite the fact that the PB dose calculation showing significant dose overestimation when compared to more‐accurate MC dose calculation, an excellent local tumor control rate (94.4% at three years) was achieved for this group of patients.^(5)^ Although our clinic practice of lung SBRT treatment changed from the PB calculation to convolution/superposition‐based algorithm, the results of this study for patients who have the longest follow‐up are important for us to understand whether the PB‐based planning contributes to the failure of local tumor control, or to the observed normal‐tissue toxicities, especially for the central tumors. For a group of patient who received SBRT but failed locally, we identified another group of patients who received the same prescription dose of SBRT and achieved local tumor control. The two groups were also matched according to the treatment intent, tumor size, histology, and follow‐up time. Our preliminary data show that overestimated PB dose calculation did not correlate with the local failures.

## V. CONCLUSIONS

In this study, we compared IMRT plans that were recalculated with the MC algorithm to clinical plans that were calculated with the PB algorithm for patients treated with SBRT. Compared with the MC calculation, significant dose overestimation of the tumor targets were found from plans calculated with PB calculation. These results are similar to the published data for patients treated with 3D conformal arc therapy with heterogeneity corrections. The magnitude of dose overestimation showed strong volume dependence for the peripheral tumors, but not for the central tumors. Despite the dose difference in tumor targets, the MC and PB calculated MLD and $V_{20}$ of the combined lungs were excellently correlated. No significant differences were found for the maximum dose for any of the other organs at risk.

## ACKNOWLEDGMENTS

This research is in part supported by a research grant from Siemens Medical Solutions.
